# Effect of Exercise on the Resting Metabolic Rate and Substrate Utilization in Women with Gestational Diabetes Mellitus: Results of a Pilot Study

**DOI:** 10.3390/metabo12100998

**Published:** 2022-10-20

**Authors:** Eleftheria Taousani, Dimitra Savvaki, Efrosini Tsirou, Maria G. Grammatikopoulou, Basil C. Tarlatzis, Dimitrios Vavilis, Dimitrios G. Goulis

**Affiliations:** 1 Unit of Reproductive Endocrinology, 1st Department of Obstetrics and Gynecology, Medical School, Faculty of Health Sciences, Aristotle University of Thessaloniki, GR-56429 Thessaloniki, Greece; 2 Department of Midwifery, Faculty of Health Sciences, International Hellenic University, Alexander Campus, Sindos, GR-57400 Thessaloniki, Greece; 3 School of Physical Education and Sports Science, Democritus University of Thrace, GR-69100 Komotini, Greece; 4 Department of Rheumatology and Clinical Immunology, University General Hospital of Larissa, Faculty of Medicine, School of Health Sciences, University of Thessaly, Biopolis, GR-41110 Larissa, Greece; 5 Medical School, University of Cyprus, 93rd Agiou Nicolaou Street, Engomi, Nicosia 2408, Cyprus

**Keywords:** energy expenditure, gestation, carbohydrate metabolism, calorimeter, pedometers, gestational weight gain, indirect calorimetry, lifestyle medicine, respiratory quotient

## Abstract

Regular physical activity during pregnancy has a positive effect on the mother and fetus. However, there is scarce data regarding the effect of exercise in pregnancies complicated by gestational diabetes mellitus (GDM). The aim of the present parallel, non-randomized, open-label, pilot, clinical study was to examine the effect of two exercise programs on the resting metabolic rate (RMR) and substrate utilization in pregnancies complicated by GDM, compared with usual care (advice for the performance of exercise). Forty-three pregnant women diagnosed with GDM between the 24th and 28th gestational week, volunteered to participate. Three groups were formed: *Usual care* (*n* = 17), *Walking* (*n* = 14), and *Mixed Exercise* (*n* = 12). The *Usual care* group was given advice on maintaining habitual daily activities without any additional exercise. The *Walking* group exercised regularly by walking, in addition to the habitual daily activities. Finally, the *Mixed Exercise* group participated in a program combining aerobics and strength exercises. Training intensity was monitored continuously using lightweight, wearable monitoring devices. The *Walking* and *Mixed Exercise* groups participated in the training programs after being diagnosed with GDM and maintained them until the last week of gestation. RMR and substrate utilization were analyzed using indirect calorimetry for all participants twice: between 27th and 28th gestational week and as close as possible before delivery. No differences were observed between groups regarding body composition, age, and medical or obstetrical parameters before or after the exercise programs. RMR was increased after the completion of the exercise interventions in both the *Walking* (*p* = 0.001) and the *Mixed Exercise* arms (*p* = 0.002). In contrast, substrate utilization remained indifferent. In conclusion, regular exercise of moderate intensity (either walking, or a combination of aerobic and strength training) increases RMR in women with GDM compared to the lack of systematic exercise. However, based on the present, pilot data, these exercise regimes do not appear to alter resting substrate utilization.

## 1. Introduction

Pregnancy is a critical period for adaptive fetal programming, with important ramifications for the health of the offspring [1,2,3,4]. Excessive gestational weight gain (GWG) increases the risk for complications for both the mother and the fetus, including pregnancy-induced hypertension [5], macrosomia, stillbirth, and gestational diabetes (GDM) [6,7,8].

GDM is the most common metabolic disorder of gestation, affecting 14% of pregnancies annually and accounting for approximately 90% of the diabetes cases during pregnancy [9,10]. Women with GDM have increased perinatal morbidity, increased risk for GDM in a future pregnancy, and increased risk for type 2 diabetes later in life [11,12]. Moreover, they have more chances of delivering babies who are classified as large for gestational age, require admission to the neonatal intensive care unit, have neonatal jaundice or respiratory distress syndrome [12,13]. The prevalence of GDM is increasing worldwide in parallel with overweight and obesity [14] and according to research, GWG consists of an independent risk factor for the development of GDM [15,16].

Lifestyle therapy, including diet and regular exercise are key components in preventing excessive GWG [17,18,19] and reducing the risk of GDM [20,21]. Exercise in particular, induces increases in the resting metabolic rate (RMR) of women and improves insulin sensitivity in the skeletal muscle [17]. Furthermore, at the molecular level, exercise triggers different pathways, all synergistically promoting greater glucose uptake by the skeletal muscles [22]. Although several studies have demonstrated the effect of physical activity (PA) in uncomplicated pregnancies, very limited data exist on its effect in pregnancies complicated by GDM. Moreover, it is unclear whether any changes in RMR are accompanied by favorable changes in substrate utilization (increased carbohydrate [CHO] use) in pregnancies complicated by GDM.

Therefore, the aim of the present study was to examine the effect of personalized aerobic and mixed types of exercise sessions on the RMR and substrate utilization of women with GDM, compared with women with GDM receiving usual care (without exercise intervention).

## 2. Materials and Methods

### 2.1. Study Details

The present study was a non-randomized, parallel, open-label, pilot clinical trial, comparing participation to personalized exercise schedule to usual care in women with GDM. Study design and findings are reported according to the Transparent Reporting of Evaluations with Non-randomized Designs (TREND) statement [23]. The protocol was registered at the Open Sciences Framework (OSF).

### 2.2. Recruitment and Inclusion Criteria

Singleton pregnant women diagnosed with GDM were recruited from the Pregnancy Complications outpatient clinic of a tertiary care university hospital, between the years 2014 and 2016. GDM diagnosis was based on the International Association of Diabetes and Pregnancy Study Group (IADPSG) [24] criteria. Inclusion and exclusion criteria for participation in the study are presented in Table 1.

### 2.3. Ethics

The study protocol was approved by the Institutional Review Board of the Medical School, Aristotle University of Thessaloniki (A7922/18-04-2011). All participants were informed of the nature and purpose of the study prior to the provision of informed consent. Subjects were able to withdraw from the study at any time, according to the Declaration of Helsinki’s ethical standards for experimentation on humans.

### 2.4. Interventions

Women interested in participating voluntarily in the study, fulfilling the study criteria (Table 1), provided consent and filled in a health history and PA questionnaire, underwent an oral glucose tolerance test (OGTT), routine blood tests, and assessment of their body mass index (BMI) between the 24th and 28th gestational week. Those with pregnancy-related complications, history of miscarriage, hemorrhage, placental abnormalities or premature contractions, were assigned to the usual care arm.

**Figure 1:**
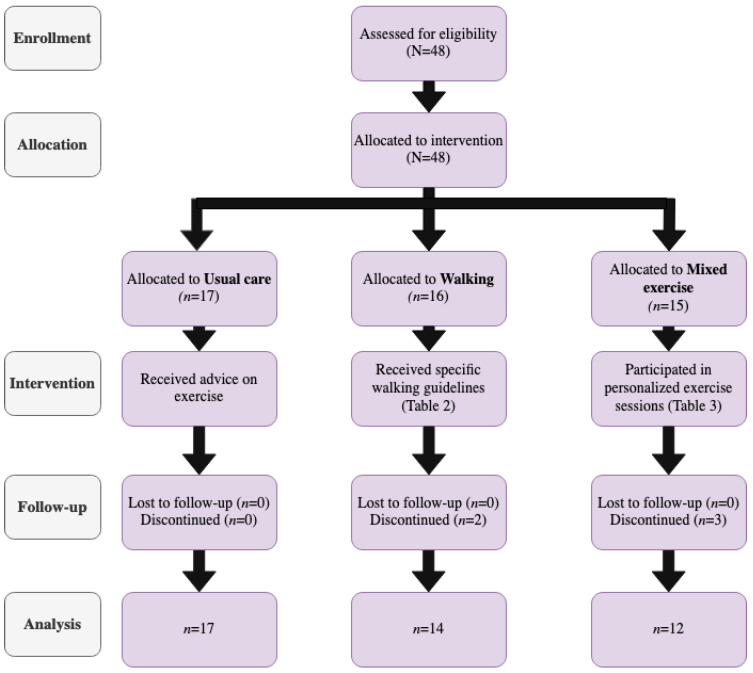
TREND [23] diagram of the study’s process.

A total of three intervention groups were formed, as detailed in Figure 1.

The first group was the *Usual care* cohort, including women with GDM who did not participate in any regular exercise program or any other strenuous PA during the study. These women received the usual advice for exercise according to the American Diabetes Association (ADA) guidelines [25,26]. The second arm involved the *Walking* group, including women with GDM who walked regularly during their daily activities/chores, fulfilling the PA levels recommended by the guidelines [27], as indicated by the PA questionnaires. Finally, the third arm consisted of the *Mixed Exercise* group, including women with a GDM diagnosis who performed a combination of aerobic and strength training programs, in one-on-one sessions with a physical education specialist (D.S.)

Specific dietary instructions were provided to all participants, based on the ADA guidelines [26,28] for a healthy pregnancy with GDM. For the *Walking* and *Mixed Exercise* arms, the additional exercise-related caloric intake during the exercise days was calculated, and additional food choices and specific instructions were given to meet the additional caloric needs [29].

Details regarding the frequency, duration, volume, intensity and type of exercise, as well as the equipment used in each session are presented in Table 2 and Table 3 for the *Walking* and *Mixed exercise* arms, respectively. Prior to each training session, women were instructed to monitor blood glucose concentrations and arterial blood pressure (BP), in order to ensure that they were within normal range. Small and lightweight wearable devices were worn during exercise to monitor exercise compliance and store exercise data (TE6000 watch and heart rate [HR] chest strap, Polar Inc., Kempele, Finland). Intensity of exercise was prescribed based on the maximum HR (HR_max_), calculated using a mathematical formula [220 − age (years)] [30].

### 2.5. Treatment Adherence

All participants were instructed to keep diaries recording their daily activities, and were advised to avoid any strenuous physical activity beyond the required one for the purpose of the study. Moreover, every other week, there was telephone contact with the participants, in order to ensure that subjects complied with the instructions.

In parallel, diaries of the nutritional intake of participants were also kept on demand, at several timepoints during the trial, aiming to assess adherence to the dietary guidelines for GDM. These diaries were checked by a registered dietitian (Ef.T.).

### 2.6. Baseline Lab Appointment

At the baseline visit (27th–28th gestational week), subjects reported to the lab following an overnight fast for the determination of their RMR. Before testing, only water consumption was allowed, and walking was kept to a minimum. Throughout the procedure, room temperature was kept constant between 19 and 23 °C and the noise was limited to a minimum to avoid destruction. Initially, body weight and height were measured using a Seca 700 mechanical scale with an attached stadiometer (Seca, Hamburg, Germany), respectively. BP was measured using a digital oscillometer (Omron HEM-907, Omron, Kyoto, Japan), with participants lying in resting state, according to manufacturer guidelines. All BP measurements were repeated thrice, with a minute’s rest in-between, and the average of the three measurements was used as mean arterial BP.

After the initial checkup, participants rested in a comfortable armchair for 5 min. The RMR of each participant was measured for 30 min through indirect calorimetry (True One Metabolic Measurement System, Parvomedics, Salt Lake City, UT, USA), following standardized procedures described previously [32]. Gas analyzers and pneumotach were calibrated prior to each RMR testing, based on the manufacturers’ instructions. The RMR was calculated according to Weir equation [33] and expressed as resting energy expenditure (REE). Substrate utilization was determined using indirect calorimetry. Total fat and CHO oxidation rates were calculated according to the non-protein respiratory quotient (RQ) [34]. Body fat, as a percent of body weight, was calculated using pregnancy-specific equations [35].

### 2.7. Second Lab Appointment

The same procedure was repeated for all women between 36th and 40th gestational week. For women grouped in the *Walking* and *Mixed Exercise* arms, this timing coincided with the end of the prescribed exercise regimen. Between baseline and second lab visits, women were visiting the outpatient clinics regularly, for the conduction of routine biochemical, hormonal, and urinary tests, clinical assessment and monitoring of fetal growth. During these visits data were downloaded from monitoring devices and dietary and exercise-related feedback was provided. Maternal and fetal-related data (gestational week, fetal weight, mode of delivery, Apgar Score) were recorded at delivery.

### 2.8. Statistical Analyses

Quantitative variables were described with central tendency and distribution measures (mean, median, mode, standard deviation), and qualitative variables with frequencies (absolute and percentage). The Shapiro–Wilk test was used to test for normal distribution. Student’s *t*-test or analysis of variance (ANOVA) were employed to test differences among groups for independent variables and paired *t*-test or repeated measures ANOVA for dependent variables. The statistical significance level was set at 5% (*p* < 0.05). Between-group comparisons for independent non-parametric data were performed using the Mann–Whitney U, or the Kruskal Wallis test. Dependent paired non-parametric data were tested using Wilcoxon signed-rank, or Friedman tests. Associations between categorical variables were tested using Chi-square or Fischer exact tests, depending on the number of variables. The Statistical Program for Social Sciences (SPSS, SPSS Inc., Chicago, IL, USA) software was used for all analyses. A *per protocol* analysis was performed.

## 3. Results

A total of forty-eight pregnant women diagnosed with GDM entered the study. Of them, 43 completed the protocol. Five women dropped out, or experienced complications, which prevented them from completing the intervention. Anthropometric, baseline characteristics and the number of participants assigned in each study group are presented in Table 4. No difference was observed among the three groups regarding the age, weight, height, and estimated fetal weight prior to participation.

Exercise data are presented in Table 5. Average exercise intensity was expressed as percent (%) of age-predicted HR_max_ and absolute HR. Weekly exercise frequency and net exercise duration were similar between participants in the *Walking* and *Mixed Exercise* arms.

REE data of each group before and after the interventions are detailed in Table 6. Mean REE did not differ among the three groups, neither at baseline (*p* = 0.791), nor at the end of the intervention period (*p* = 0.694). However, when the difference before and after the intervention within each group was calculated, participants in the *Walking* (*p* = 0.001) and the *Mixed Exercise* group demonstrated an increased REE post-intervention (*p* = 0.002). Regarding the changes in CHO utilization post-intervention, mean CHO energy expenditure did not differ among the three groups, neither at baseline (*p* = 0.853) nor at the end of the intervention (*p* = 0.698). When changes prior to and post-intervention within each arm were examined, no differences were observed.

## 4. Discussion

The present study aimed to examine the effect of aerobic and mixed exercise types on the RMR and substrate utilization in women with GDM compared with women on usual care. The results showed that regular aerobic or mixed exercise conducted between the 27th and 38th gestational weeks increases the RMR of women with a GDM diagnosis compared to those who do not exercise. These effects were observed when exercise was performed three times weekly, for a duration of 40 min in each session, at an exercise intensity of 68% of age-predicted HR_max_. With regard to substrate utilization, the prescribed exercise (aerobic or mixed) did not appear to alter CHO metabolism in women with GDM compared to those not exercising.

Exercise prescription during pregnancy is a difficult task, as the majority of childbearing women tend to retain a sedentary lifestyle and are low adherers to the exercise recommendations [36,37,38,39]. Similar findings have also been reported for women with GDM [40]. Although most women receive some form of exercise advice from their gynecologists, it appears that the latter do not have the necessary training, knowledge, or support to provide specific exercise advice for gestation [41]. Therefore, the lack of an effect of PA recommendations on the RMR of pregnant women herein appears justified, given that despite the advice provided as usual care, most women tend to remain sedentary. Previous research has shown that often, women are discouraged by their environment to be physically active during pregnancy, with many being told to stop any form of exercise for the health of the baby [38]. Moreover, changes in body weight, pregnancy complications, and external factors affect the levels of PA during pregnancy, inducing a great variability in PA over the course of gestation [42].

Throughout pregnancy an increase in RMR and a concomitant decrease in activity energy expenditure is observed [32,40,43,44]. These changes are more profound during the third trimester of gestation, as a result of higher body mass accumulation [44]. Women with GDM also exhibit greater energy requirements due to their greater body mass, however, restricting energy intake is recommended by most scientific organizations in order to limit GDM- and obesity-related adverse events [28]. The present study revealed that regular aerobic or mixed type exercise, performed between the 27th and 38th gestational weeks increases RMR in women with GDM complicated pregnancies. This finding is *in* lieu with a recent systematic review [45], recommending that women with GDM should exercise for at a least moderate intensity, twice weekly of more frequently, for approximately 20–50 min in order to benefit from the performed PA. During pregnancy, REE is greatly dependent on body mass, body composition, and other anthropometric indices, including abdominal circumference and fetal-specific characteristics [46,47]. Since fat-free mass is metabolically active [48], greater fat-free mass is associated with higher REE throughout the life cycle, including during pregnancy [49,50,51]. With the observed variations in RMR during gestation being greatly associated to the changes in body composition [49,50], it becomes clear that any level of exercise inducing changes in the body composition may, in fact, alter RMR. Moreover, according to a recent Japanese study [49], REE of childbearing women with good glycemic control tends to be lower than that of women with poorer glycemic control. Thus, it appears that other factors might also interplay with REE in this population, limiting the effects of exercise.

Although regular exercise increased RMR among women with GDM, it did not appear to alter substrate utilization at a resting state. Overall, compared to women with uncomplicated pregnancies, those with GDM demonstrate a gradual reduction in insulin sensitivity during gestation, as a possible residue of the accumulated body mass [52]. Among women with GDM, during the last trimester of pregnancy REE is highly correlated to the abdominal circumference of the mother, as well as to the birthweight of the offspring [46]. With regard to exercise, researchers have reported increased CHO utilization in pregnant women during the performance of acute PA [53] and similar findings were also reported in individuals with diabetes mellitus [54], both indicative of a reduced efficiency in carbohydrate use. However, according to the present findings, no difference is observed with regard to CHO utilization between sedentary and exercising pregnant women with GDM, at a resting state. It is possible that the GDM diagnosis and the dietary and medication use to maintain optimal glycose concentrations at resting state, might have induced similar improvements in the substrate utilization. Medication and/or diet may have a stronger effect on CHO metabolism compared with exercise. Another explanation could be that the prescribed exercise intensity was insufficient to affect resting CHO utilization in women with GDM, since increased exercise intensity increases glycogen use. Furthermore, this was a pilot study and, in this manner, it is possible that the small number of recruited women did not allow for substantial changes in the RMR post-exercise.

In the present study, the exercise training programs increased RMR but failed to affect resting CHO use in women with GDM. Since participation in PA increases CHO utilization during the day, it may also reduce the need for insulin use. Alterations in the components of the prescribed exercise training may reveal a more effective exercise prescription for improved glucose regulation in women with GDM. Since insulin resistance is greatest in the third trimester and screening for GDM usually occurs around 24th–28th gestational week, the length of the training program cannot be altered or extended. Therefore, future research should focus on increasing intensity (>70% of age-predicted HR_max_) and/or training duration (>40 min, >3 times/w), in order to examine for potentially favorable effect on resting substrate utilization.

Since the year 2002, a total of 11 international organizations have developed clinical practice guidelines for exercise in uncomplicated pregnancies [27], all recommending the performance of moderate intensity exercise. Given that the positive effect of exercise relates more to the daily energy expenditure than to the use of CHO, moderate exercise may not necessarily improve glucose concentrations. Therefore, tight blood glucose control should be maintained in women with GDM who begin exercise during the third trimester. This study’s findings and data from other studies can be used to develop a consensus for exercise guidelines in GDM.

The strengths of the present study include the strict study protocol, the control of medical care (diet, blood glucose, medications), and the close monitoring of all exercise sessions by experienced supervisors. In addition, modern wearable devices and personal contact were used for the *Walking* group, while exercise for the *Mixed Exercise* group was delivered and monitored by the same exercise professional every time, which allowed for the tight control of the exercise-related variables.

The main limitation of the present study involves the lack of randomization of participants in the three intervention arms. Nevertheless, specific rules were followed for the assignment of the women. First, women with contraindications for exercising were assigned exclusively to the *Usual care* arm. Second, women who express unwillingness to participate in the *Mixed Exercise* arm (usually due to lack of time) were properly randomized (sealed, opaque envelop method) into one of the other two groups. Third, women who agreed to participate in any of the three groups were properly randomized (sealed, opaque envelop method) into any group, including the *Usual care* arm. Another limitation is that, although subjects were monitored closely during exercise programs, we relied on what they reported concerning their activity during the rest of the day. Fourth, we did not measure true HR_max_ for intensity prescription, but instead, it was calculated through a mathematical formula. Therefore, the possibility that some subjects may have had different HR_max_ from what was calculated cannot be ruled out, resulting in these individuals exercising at different exercise intensities from the assigned protocol.

With regard to the method used for the estimation of energy requirements, indirect calorimetry (IC) consists of the most widely used method for RMR determination and has been found to be consistent and in close agreement with direct calorimetry [55,56]. IC is non-invasive, producing minimal subject discomfort, and it also allows determination of the substates that are being used [55,57]. Although for some scientists IC consists of the gold standard method for the evaluation of energy needs in clinical practice [57], it still entails few limitations. For instance, IC requires a facemask/mouthpiece to be applied, a fact that may induce a stress response is some subjects, prior to and during the testing procedure [55]. Furthermore, movements must be kept to the minimum and air leakages in the respiratory circuit may affect results [55,56].

## 5. Conclusions

In conclusion, regular, moderate-intensity exercise, either in the form of walking only, or mixed exercise type (aerobic and strength combined) performed between the 27 and 38th gestational week, increases RMR in women with a GDM diagnosis compared to the usual care (advice to perform exercise). However, the performed exercise does not appear to affect CHO utilization at a resting state in women with GDM. Nonetheless, the present study was a pilot one, aiming to provide initial information on the RMR of women with GDM. In this manner, the present findings could serve as a starting point, without however, undermining the need for more research, in order to determine the optimal exercise program for women with GDM, according to their individual characteristics (precision exercise).

## Figures and Tables

**Table 1:** Exclusion and exclusion criteria.

Inclusion Criteria (All Groups)	Exclusion Criteria (All Groups)
Diagnosis of GDM based on the IADPSG criteria: abnormal OGTT following intake of 75 g glucose between 24th–28th gestational week. Singleton pregnancy BMI 18.5–40.0 kg/m^2^ Age < 40 years Gestational week < 30 Regular PA < 2 times per week, during the past 6 months	Pre-existing diabetes mellitus (type 1 or 2) Smoking (any amount) Alcohol intake (any amount) Uncontrolled thyroid disease Chronic renal or liver dysfunction Cancer Use of CCs, progesterone, or chemotherapeutic agents Diagnosis of any disease (excluding GDM) during pregnancy
	Exclusion criteria (*Walking* and *Mixed exercise* arms only)
	History of miscarriage Premature contractions History of hemorrhage History of placental abnormalities

BMI, body mass index; CCs, corticosteroids; GDM, gestational diabetes mellitus; IADPSG, International Association of Diabetes and Pregnancy Study Groups [24]; OGTT, oral glucose tolerance test; PA, physical activity.

**Table 2:** *Walking* arm guidelines.

Exercise mode *:	Walking (outside) with mean ascend 0–1%.
Warm-up *:	Stretching followed by 5–10 min walking (self-selected pace 40–50% of HR_max_), followed by a 2–3 min rest.
**Frequency** (**times**)**:**	At least 3 times weekly.
**Duration** (**min**)**:**	Between 30 and 45 min for the main part, plus 15–20 min for warm-up and cool-down.
**Intensity** (**% of HR_max_**) **^†^:**	Self-selected between 60 and 70% of age-predicted HR_max_. Individual upper and lower HR limits were set, using an audio signal.
**Pace:**	Self-selected pace within the above intensity and duration limits.
Cool-down *:	5 min slow-paced walking and 5 min stretching.

bpm, beats per minute; GPS, Global Positioning System; HR, heart rate; HR_max_, maximum heart rate; min, minutes; **^†^** the intensity zone was set at 60–65% during week 1, and 60–70% thereafter; * all activities took place outdoors, to allow GPS signal and monitoring. Data were automatically stored during the training session, from the wearable devices. Each week, data were downloaded and analyzed. Parameters recorded during exercise included exercise frequency, duration (min), total distance (m), ascend distance (m), mean and maximum heart rate (bpm), mean and max walking speed (km/h) [using a Polar TE6000 watch and HR chest strap (Polar Inc., Kempele, Finland)] and the number of steps [using pedometer].

**Table 3:** *Mixed-exercise* arm guidelines.

**Exercise modes ^¥^:**	Treadmill walking (indoors) Muscle strength using bodyweight, fitball, and dumbbells		
**Frequency** (**times/w**)**:**	3 times/w distributed as follows: - 1 d/w walking only - 1 d/w muscle strength only - 1 d/w combination of walking and muscle strength training		
**Duration** (**min**)**:**	Between 50–60 min/session (including warm-up and cool-down)		
**Intensity** (**% of HR_max_**)**:**	As described below		
**Equipment:**	Treadmill: Excite Medical (Technogym^®^, Cesena, Italy)		
**Treadmill walking**			
***Content***		*Intensity*	*Duration*
**Warm-up:**	Walking	50% HR_max_	5 min
**Main part:**	Walking	60–70% HR_max_ *	20 min
**Cool-down:**	Walking	30% HR_max_	5 min
	Stretching		15 min
**Strength training**			
***Content***		***Intensity***	***Duration***
**Warm-up:**	Walking	50% HR_max_	5 min
	Active Stretching		5 min
**Main part:**		13–14 points on the Borg RPE Scale (0–20)	30 min
**Strength exercises:**	Knee extension (quads) Knee flexion (hams) Arm curl (biceps) Triceps pull (triceps) Shoulder raise (deltoid) Leg adduction (Adductors) Leg abduction (Abductors) Half-squats	2–3 sets × 15 reps for all strength exercises ^†^	
**Cool-down:**	Walking	30% HR_max_	5 min
	Stretching		15 min
**Combined walking and strength training**			
***Content***		***Intensity***	***Duration***
**Warm-up:**	Walking	50% HR_max_	5 min
	Active stretching		5 min
**Main part:**	Walking	60–70% HR_max_	15 min
	Strength training:	13–14 points using the Borg RPE Scale (0–20)	15 min
	Strength exercises:	1 set × 15 repetitions ^‡^	
**Cool-down:**	Walking	30% HR_max_	5 min
	Stretching		15 min

bpm, beats per minute; d, day(s); HR, heart rate; HR_max_, maximum heart rate; min, minutes; RPE, Rating of Perceived Exertion using the Borg scale [31]; w, week(s); * during the first week, the intensity was set at 60–65% of HR_max_ and at 65–70% thereafter; ^†^ during week 1, two sets were performed, and three sets after that. Between exercises, rest time was set at 1 min and between sets at 3 min; ^‡^ between exercises, rest time was set at 1 min and between sets at 3 min; ^¥^ all sessions took place indoors. Walking was performed first, followed by strength exercises when both walking and strength training were included. The same qualified personal trainer was always present, giving instructions, monitoring exercise training, and providing feedback and/or corrective actions. Parameters recorded during exercise included exercise frequency, duration (min), mean and maximum HR (bpm), RPE, mean and maximum walking speed (km/h), using a Polar TE6000 watch and HR chest strap (Polar Inc., Kempele, Finland).

**Table 4:** Anthropometric and baseline characteristics * during the first antenatal visit.

Variabes	*Usual Care* (*n* = 17)	*Walking* (*n* = 14)	*Mixed Exercise* (*n* = 12)	*p*-Value
Maternal age (years)	33.4 (31.1–35.7)	35.1 (32.6–37.6)	34.0 (31.3–36.7)	0.571
Maternal height (cm)	167.5 (164.5–170.7)	165.6 (162.3–168.9)	166.2 (162.3–168.2)	0.475
Maternal weight (kg)	86.4 (78.3–94.5)	88.1 (78.9–97.3)	77.8 (70.0–85.7)	0.177
Estimated fetal weight (g)	1998 (1744–2250)	1847 (1483–2209)	1598 (1165–2030)	0.296

* Values are expressed as mean and the respective 95% confidence intervals.

**Table 5:** Exercise program characteristics * for the *Walking* and *Mixed Exercise* arms.

Variables	*Walking* (*n* = 14)	*Mixed Exercise* (*n* = 12)
Weekly frequency	3.0	3.0
HR (bpm)	119.5	120.9
HR_max_ (bpm)	136	138
% of age-predicted HR_max_	68.3%	68.7%
Duration (min)	39	30–40 ^†^
Number of steps (*n*)	4819	-
Walking speed (km/h)	4.3	-
Walking distance (km)	2.8	-

bpm, beats per minute; HR, heart rate; HR_max_, maximum heart rate; min, minutes; * Values are expressed as mean and the respective 95% confidence intervals. ^†^ Depending on the type of exercise program.

**Table 6:** Difference in the REE (kcal/d) and CHO energy utilization (kcal/d) of participants in each arm, before and after the interventions ^†^.

Variables		*Usual Care* (*n* = 17)	*Walking* (*n* = 14)	*Mixed* *Exercise* (*n* = 12)	*p*-Value ^1^
**REE**	After − Before exercise	30.8 (−121.5 to 183.0)	243.1 * (128.5 to 357.7)	264.1 * (109.8 to 418.4)	0.026 *
	*p*-value ^2^	0.671	0.001 *	0.002 *	
**CHO** **utilization**	After − Before exercise	65.7 (−113.5 to 244.9)	138.3 (−132.5 to 409.0)	77.5 (−386.6 to 541.7)	0.907
	*p*-value ^2^	0.715	0.691	0.171	

CHO, carbohydrate; REE, resting energy expenditure; ^†^ values are expressed as mean and the respective 95% confidence intervals; ^1^ Between-group comparisons; ^2^ within-group comparisons; * *p*-value < 0.005.

## Data Availability

The data presented in this study are available on request from the corresponding author (DGG). The data are not publicly available due to privacy.
